# Context effects on object recognition in real-world environments: A study protocol

**DOI:** 10.12688/wellcomeopenres.17856.1

**Published:** 2022-05-26

**Authors:** Victoria I. Nicholls, Benjamin Alsbury-Nealy, Alexandra Krugliak, Alex Clarke

**Affiliations:** 1 Department of Psychology, University of Cambridge, Cambridge, CB2 3EB, UK; 2 Department of Psychology, University of Toronto, Toronto, M5S 3G3, Canada

**Keywords:** Object recognition, context, mobile EEG, augmented reality, real-world neuroscience

## Abstract

**Background:** The environments that we live in impact on our ability to recognise objects, with recognition being facilitated when objects appear in expected locations (congruent) compared to unexpected locations (incongruent). However, these findings are based on experiments where the object is isolated from its environment. Moreover, it is not clear which components of the recognition process are impacted by the environment. In this experiment, we seek to examine the impact real world environments have on object recognition. Specifically, we will use mobile electroencephalography (mEEG) and augmented reality (AR) to investigate how the visual and semantic processing aspects of object recognition are changed by the environment.

**Methods:** We will use AR to place congruent and incongruent virtual objects around indoor and outdoor environments. During the experiment a total of 34 participants will walk around the environments and find these objects while we record their eye movements and neural signals. We will perform two primary analyses. First, we will analyse the event-related potential (ERP) data using paired samples t-tests in the N300/400 time windows in an attempt to replicate congruency effects on the N300/400. Second, we will use representational similarity analysis (RSA) and computational models of vision and semantics to determine how visual and semantic processes are changed by congruency.

**Conclusions:** Based on previous literature, we hypothesise that scene-object congruence would facilitate object recognition. For ERPs, we predict a congruency effect in the N300/N400, and for RSA we predict that higher level visual and semantic information will be represented earlier for congruent scenes than incongruent scenes. By collecting mEEG data while participants are exploring a real-world environment, we will be able to determine the impact of a natural context on object recognition, and the different processing stages of object recognition.

## Introduction

Our visual environment has a powerful impact on many cognitive processes, one of which is the ease of, and ability to, recognise objects. Recognition is thought to be both faster and more accurate when objects appear in expected environmental contexts, compared to when they are situated in unexpected places ( Bar, 2004; Biederman *et al*., 1982; Brandman & Peelen, 2017; Cornelissen & Võ, 2017; Davenport & Potter, 2004; Henderson *et al*., 1999; Loftus & Mackworth, 1978; but see Spaak *et al*., 2022). This impact of contextual consistency is further seen in neural responses, with modulations of N300/N400 electroencephalography (EEG) components ( Draschkow *et al*., 2018; Ganis & Kutas, 2003; Kovalenko *et al*., 2012; Lauer *et al*., 2018; Lauer *et al*., 2020; McPherson & Holcomb, 1999; Mudrik *et al*., 2010; Mudrik *et al*., 2014; Võ & Wolfe, 2013), often linked to the access of semantic knowledge ( Kutas & Federmeier, 2011). While these studies establish that visual contexts impact the processing of objects, they have done so using carefully controlled paradigms and situations, which necessarily involved removing the participant from *‘the wild’,* instead presenting participants with visual depictions of the world. There is an increasing trend towards studying cognition in more naturalistic settings ( *e.g.*, Aliko *et al*., 2020; Hamilton & Huth, 2020; Sonkusare *et al*., 2019), facilitated by advances in mobile imaging that allow for neural recordings in freely moving participants ( *e.g.*, Delaux *et al*., 2021; Krugliak & Clarke, 2022; Ladouce *et al*., 2019; Reiser *et al*., 2019). Examining cognitive faculties in an ecologically valid manner is important, as it’s known that some fundamental neural properties – the selectivity of orientation cells in V1 ( David *et al*., 2004) and place cells in the hippocampus ( Aghajan *et al*., 2015) – change between controlled and natural environments. Further, neuroimaging effects do not always translate between seeing pictures of objects, and seeing the real objects themselves ( Snow *et al*., 2011). This underlines the importance of research conducted in situations that more closely mirror our everyday interactions with the world.

**Figure 1.**
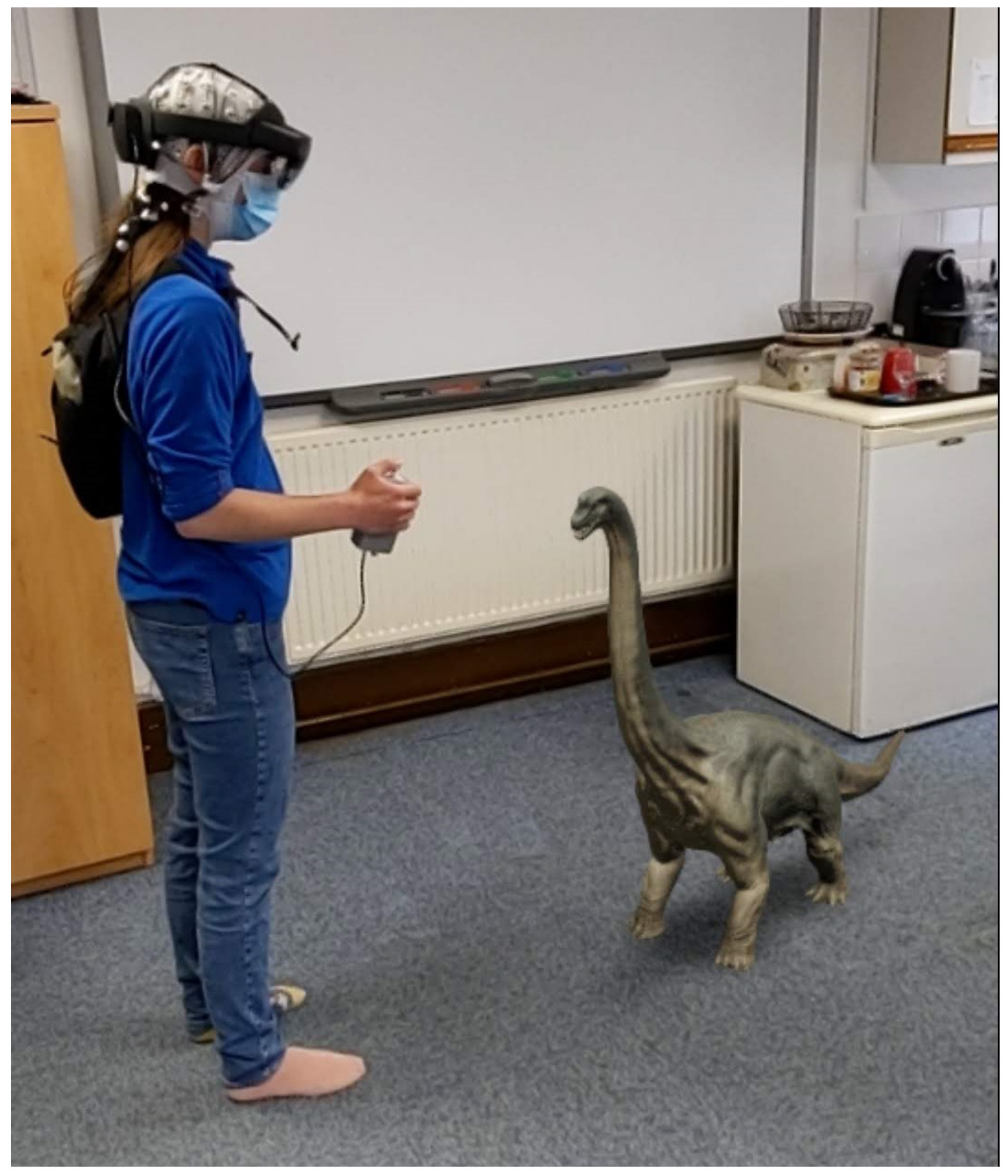
Example apparatus setup on one of the authors with an example AR object. AR, augmented reality.

In this research, we first seek to examine the impact real-world environments have on object recognition with mobile EEG (mEEG). To understand object recognition in real-world environments, we will utilise recent advances in augmented reality (AR) technology to display virtual objects that are embedded into the environment, creating a mixed reality experience ( Figure 1). This approach allows us to record neural signals while participants are immersed in real-world environments, whilst also controlling the objects that are seen using an AR headset ( Krugliak & Clarke, 2022). Such an approach can be utilised to determine if contextual effects seen in the lab translate to natural settings.

The second aspect of this research aims to test how the visual and semantic processing of objects is changed by the environment. Whilst contextual congruency effects are established in the lab, we know little about how our knowledge of where we are in the world impacts the recognition of an object when outside of the lab. For objects isolated from the background, or removed from a continuous spatiotemporal narrative, recognition is thought to involve a largely feedforward propagation of signals along the ventral visual pathway within 150ms, supporting low and mid-level visual analysis of the object ( Cichy *et al*., 2016; DiCarlo *et al*., 2012; Lamme & Roelfsema, 2000). Beyond approximately 150–200ms, more complex visual and semantic information is seen, driven by recurrent dynamics in the ventral temporal lobe ( Bankson *et al*., 2018; Chan *et al*., 2011; Clarke *et al*., 2011; Clarke *et al*., 2018; Poch *et al*., 2015). However, as these findings are largely based on experiments where the object is isolated from an environment, either in the sense that the item is presented without a spatial context, or where the item is in context, but the natural spatiotemporal structure is broken, we know little about what aspects of the recognition process are modulated by context. Given contextual congruency impacts the N300/N400 components ( Draschkow *et al*., 2018; Ganis & Kutas, 2003; Kovalenko *et al*., 2012; Lauer *et al*., 2018; Lauer *et al*., 2020; McPherson & Holcomb, 1999; Mudrik *et al*., 2010; Mudrik *et al*., 2014; Võ & Wolfe, 2013), we predict that semantic processes will be impacted, however, to get a full picture of which aspects of the recognition process are impacted and when, we need to test how the representational dynamics of visual and semantic processes shift depending on contextual congruency of the object. Here, we seek to test this using a novel application of representational similarity analysis ( Kriegeskorte *et al*., 2008) to mEEG signals during the perception of virtual objects.

The current protocol aims to further develop methods and approaches for mEEG and combine this with emerging AR technology to effectively study real-world recognition, and how the context impacts the processing of the visual and semantic object properties.

## Protocol

### Participants

A total of 34 young adult participants (18–35 years old) with no history of neurological conditions, and with normal or corrected-to-normal vision will be recruited from the University of Cambridge and local areas. Participants will be compensated at a rate of £15/hour for their time. A sample size of 34 was determined using power analyses on data from Draschkow *et al*. (2018) that examined congruency effects on object-scene processing. This was calculated using the ‘sampsizepwr()’ function in MATLAB ( MATLAB, 2021, RRID:SCR_001622), based on the mean difference between the amplitude of the N400 on congruent and incongruent conditions, to obtain a power of 0.8, at an alpha of 0.05. This study has been approved by the Department of Psychology ethics committee at the University of Cambridge (PRE2020.007, date of approval: 08/04/2020). Written informed consent will be obtained from all participants prior to taking part in the study. The study will be performed in accordance with all appropriate institutional and international guidelines and regulations, in line with the principles of the Declaration of Helsinki.

### Apparatus

Participants will be presented with AR stimuli using a Hololens 2 device. The Hololens 2 has a horizontal field of view of 42°, a vertical field of view of 29°, and presents images at 60Hz. Eye movements will be tracked using the Hololens 2 with a sampling rate of 30Hz, average gaze position error of about 1.5°. Eye tracking calibration will be performed using a nine-point calibration procedure. EEG will be recorded using the Brainvision LiveAmp 64 mobile system (Brain Products GmbH, Gilching, Germany, RRID:SCR_009443). In all sessions we will record 64-channel EEG through ActiCap Slim active Ag/AgCl electrodes, with a reference electrode placed at FCz and a sampling rate of 500Hz. The EEG electrodes are placed on the participant with the Hololens 2 placed on top of the electrodes, and the LiveAmp and electrode cables placed in a backpack that the participant wears while performing the experiment. A custom-built button box is plugged into the Hololens 2 USB-C port and the LiveAmp trigger port, meaning that when the button is pressed, it simultaneously sends a signal to Hololens 2 and a 5V signal to the LiveAmp. The LiveAmp then converts that signal to a trigger that is marked in the EEG recording. The signal to the Hololens 2 triggers the appearance of an object. An example of the setup can be seen in Figure 1.

### Stimuli and procedure

In this experiment, participants will walk through two different environments, one indoor environment and one outdoor, where they will see virtual objects in various locations. A total of 85 images of concepts from a property norming study will be used ( Hovhannisyan *et al*., 2021). Five of the 85 images will be presented in the practice trials, and the remaining 80 will be presented in the main experiment. The stimuli will be placed in the environment using Experimenter (beta testing version), an experiment design software package built for the Unity engine and presented to participants with the Hololens 2. A list of the stimuli used and their locations in the experiment can be found as *Extended data* ( Nicholls, 2022).

The 80 images presented in the main experiment will be presented in four blocks of 20 images. The images in each block will be matched on the following predictors: congruency, environment, category, domain, hit rate on a visual task, hit rate on a lexical task, false alarm rate for a lexical task, false alarm rate for a visual task, image energy, image size, proportion of non-white space in the image, hue of the image, saturation of the image, frequency in COCA database, proportion of participants identifying the modal name of the object, and the number of non-taxonomic features. With the exception of congruency and environment, the predictors for each image will be taken from Hovhannisyan *et al*., 2021. The matching will be done using the Match software (version 2.09.02, Van Casteren & Davis, 2007).

**Figure 2.**
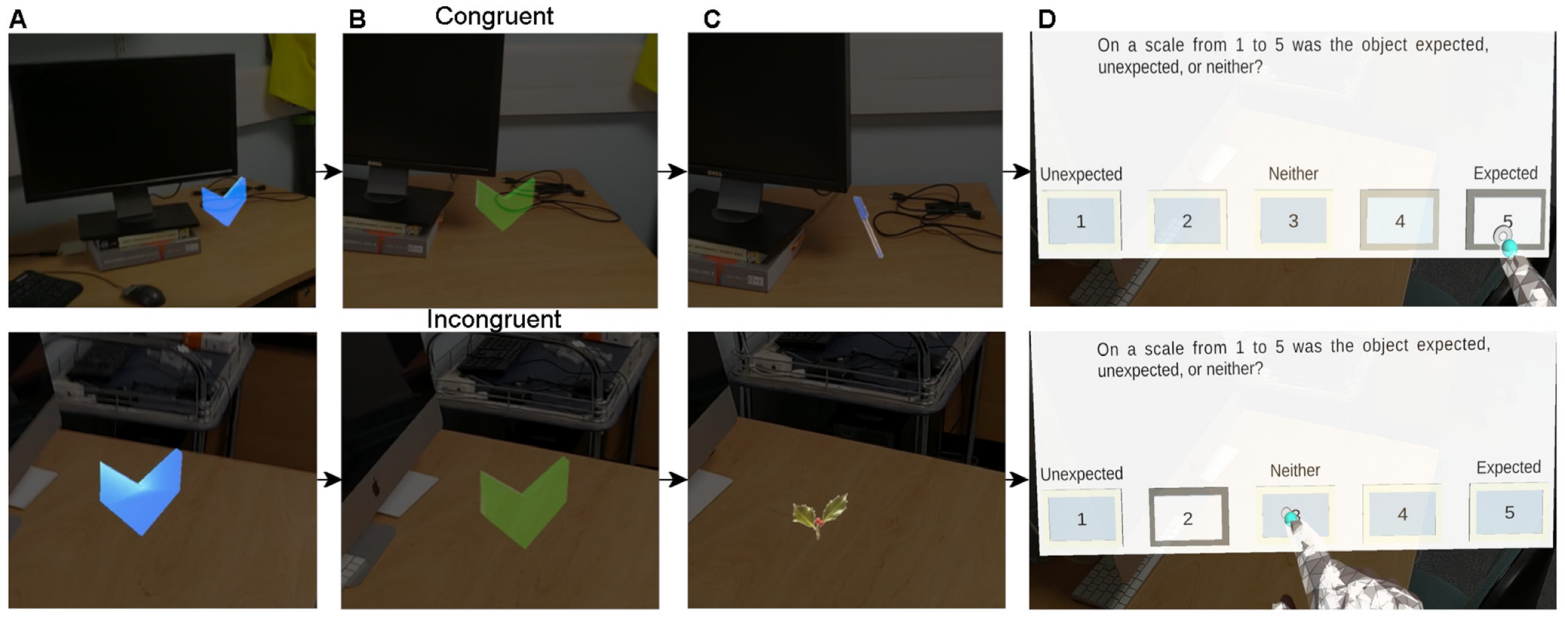
Trial protocol. ( **A**) Example blue arrow that participants will search for. ( **B**) When participants are close enough to the blue arrow it will turn green indicating that they need to press the button on the button box. ( **C**) Example indoor trials. For ( **A**), ( **B**), and ( **C**) the top panel shows an example of a trial with a congruent object, and the bottom panel a trial with an incongruent object. ( **D**) An example of the question that will be shown to participants when the object disappears.

To familiarise the participants with the Hololens and the appearance of AR stimuli they will first perform five practice trials in an indoor environment. The trials follow the same procedure as for the experiment described below. During the experiment participants will walk through two different environments, one indoor environment, one outdoor environment. In each environment participants will complete two blocks of 20 trials (40 total per environment). In each trial participants will need to find an arrow indicating the location of the object ( Figure 2A). When participants are close enough to the arrow, the arrow will change colour ( Figure 2B) indicating that the participants can press the button on their button box ( Figure 1). Once the button is pressed an object will appear for five seconds ( Figure 2C). At the same time a trigger is sent to the LiveAmp. Participants will be instructed to look at the object for the entire time it is visible, and to keep as still as possible. After the object disappears participants will be presented with a question, asking them how expected the appearance of the object was on a scale from one to five ( Figure 2D). One is unexpected, three is neither, and five is expected. Once they have responded the next trial will begin. This process is repeated until all 80 objects are found. A total of 40 objects will be congruent with the environment, and 40 objects will be incongruent. During this time participants' EEG activity, and eye movements will be recorded.

### EEG preprocessing

For the preprocessing of the EEG data we will use the BeMoBIL pipeline ( Klug *et al*., 2018) in EEGLAB (RRID:SCR_007292, version 2021.1, Delorme & Makeig, 2004) with the example configuration.

To start with we will run the ‘bemobil_process_all_EEG_preprocessing()’ function, which downsamples the data to 250Hz, and removes frequency artefacts using the ZapLine toolbox ( de Cheveigne, 2020). The ‘bemobil_process_all_EEG_preprocessing()’ function will then find, and spherically interpolate, bad channels using ‘clean_artifacts()’ and ‘pop_interp()’ before re-referencing the data to the common average.

We will then bandpass filter the data using a Blackman filter with limits between 1 and 20Hz, with a transition width of 0.1Hz. The data will then be epoched to -2 and +2 seconds of the appearance of the object, and baseline corrected to the data between -100ms and 0ms in relation to the appearance of the object.

Once the data is epoched and baseline corrected we will run the ‘bemobil_process_all_AMICA()’ function from the BeMoBIL pipeline. This function applies a high-pass zero-phase Hanning window FIR filter with a cutoff at 1.75Hz to the data, before decomposing the data into statistically independent components using AMICA ( Palmer *et al*., 2008), and dipoles will be fitted for each component. After this, the ‘bemobil_process_all_AMICA()’ function will copy the independent components onto the bandpass filtered and epoched data (the data inputted to the function, prior to highpass filtering) and reject any components not originating from the brain, such as eye movements, using the IClabel algorithm.

Once this is complete, we will transform the data to scalp surface Laplacian, or current source density estimate using the CSD toolbox ( Kayser & Tenke, 2006). This is a reference-free estimate of the scalp current density that minimises the effect of volume conduction to increase the spatial localisation relative to electrical scalp potentials ( Nunez & Srinivasan, 2006).

### Statistical analysis of EEG data

EEG analysis of the data will be done in two parts, the first part will investigate the ERP data for congruency effects. The second will determine the impact of congruency on visual and semantic processes using representational similarity analysis (RSA).

***ERP analyses.*** For the ERP analysis we will also split this into two parts. The first part will determine whether we can replicate previous N300/400 congruency effects. To do this we will perform the same ERP analysis as in Draschkow *et al*. (2018). We will calculate the mean amplitude for two consecutive time windows: 250–350ms (N300), and 350–500ms (N400), across mid-central electrodes (electrodes FC1, FC2, C1, Cz, C2, CP1, CPz, and CP2). We will do this by averaging across trials, electrodes, and time points within subjects, separately for congruent and incongruent trials, and assessing differences with paired sample t-tests in the N300 and N400 time windows.

The second part will be to perform an exploratory analysis to investigate congruency effects across all electrodes, and from zero to 1000ms after the presentation of the object image. We will do this using hierarchical linear modelling with LIMO EEG (LIMO EEG, RRID:SCR_009592, Pernet *et al*., 2011). LIMO analyses the data in two steps: the first level consists of estimating parameters of a general liner model (GLM) for each subject at each time point and each electrode individually. The second level of the analysis takes the beta coefficients obtained from each subject in the first level of the analysis and analyses them across subjects to test for statistical significance. LIMO then applies a bootstrap cluster correction for multiple comparisons. The second level of analysis offers five statistical test options that can be performed on the beta coefficients across subjects. Here we will use a regression with a fixed factor of congruency.

***RSA analysis.*** The second part of the analysis will use RSA and computational models of vision and semantics ( Clarke *et al*., 2018) to ask how visual and semantic processes are changed by congruency, by comparing the size and latency of RSA effects between congruent and incongruent conditions. Each object is associated with EEG activity at the 64 electrodes that can be characterised as a spatial pattern of neural activity, which varies over time. For each time point within the epoch of interest (0 to 1000ms), we will calculate the dissimilarity between activity patterns for each pair of objects using 1-Pearson’s correlation. This will result in an 80×80 dissimilarity matrix showing how each object is more or less similar to each other object. These representational dissimilarity matrices (RDMs) will be calculated at each time-point.

To determine whether the RDMs capture patterns associated with visual or semantic processes, the EEG-based RDMs are subsequently correlated with model RDMs based on visual and semantic properties. The RDMs based on visual properties will be extracted from an artificial neural network model, CORnet ( Kubilius *et al*., 2019). Node activations for the 80 objects will be extracted from the visual stimuli using THINGSvision ( Muttenthaler & Hebart, 2021) and RDMs will be constructed for model layers ‘V1’, ‘V2’, ‘V4’ and ‘IT’. The RDM for semantic information will use the semantic feature lists from a concept property norming study that used the same images ( Hovhannisyan *et al*., 2021). The property norming study contains lists of object-feature pairs with 995 objects and 5520 features. Each object has a feature vector of length 5520, indicating if each feature is associated with the object. The semantic feature RDM is constructed from the dissimilarity between feature vectors for the 80 objects.

The visual and semantic RDMs (n=5) will be correlated with the EEG-based RDMs at each time-point using Spearman’s correlation giving a correlation time-course for each model RDM, and for each participant. Significant positive effects of each model RDMs will be established using one-sample t-tests against zero (p < 0.01, alpha 5% controlling for five model RDMs) and cluster-based permutation testing ( Maris & Oostenveld, 2007) to control for multiple comparisons over time. This analysis will be conducted for (1) all 80 objects together, (2) 40 congruent object-context trials and (3) 40 incongruent object-context trials.

Statistical comparisons between the congruent and incongruent RSA effects will use paired t-tests and cluster-based permutation testing. Differences in onset and peak times will be statistically tested using 1000 bootstrap resamples to create a distribution of onset and peak times for each condition. Onset latencies and distributions will be calculated as the first timepoint with a significant p-value in a one-sample t-test against zero (α=0.01) for each resampled dataset. To evaluate potential differences in the peak latencies between conditions, group average RSA time-courses will be calculated for each resampled dataset, and the timepoint of the maximum effect will be extracted, creating a distribution of peak times for each model RDM and condition. The distribution of peak and onset latencies will then be used to define 95% confidence intervals (CIs) of pairwise differences.

### Eye tracking preprocessing

Eye movements will be parsed into fixations, and saccades with a manually set velocity threshold starting at 30°/s to match the SR Research (2007) thresholds, increasing to a maximum of 80°/s if the first threshold is too low for the participant. The velocities will be changed at an individual level to adapt to the differing levels of noise present in different participants. This noise can be caused by factors related to the stability of the participant’s eye, or factors related to the stability of the recorded eye movement signal such as dryness of the eye, makeup, or occlusion due to the eyelid, reflections from glasses, etc.

We will define fixations based on the gaze direction. If the head rotates relative to our objects, but the direction of gaze stays constant we would count that as a fixation.

### Statistical analysis of eye tracking data

To get an indication of how long participants process the objects, and how this is impacted by congruency we will analyse participants’ fixation durations. We will do this using linear mixed models with a fixed effect of congruency, and random intercepts of participant and item. We will start with random slopes of congruency. In case the model does not converge we will prune the random effects model using the procedure described in Bates *et al*., 2015.

### Hypotheses

Based on the previous literature, we hypothesise that scene-object congruence would modulate object recognition, as seen in ERPs and RSA. For ERPs, we predict a congruent effect in the N300/N400 time windows, and for RSA we predict that higher-level visual and semantic information will be represented earlier for congruent scenes than incongruent scenes. For the eye tracking, we predict that participants will fixate incongruent objects for longer than congruent objects.

## Dissemination of findings

The study will be published in an open-access peer reviewed journal and the associated data and scripts for the study will be placed in an open access data repository.

## Discussion

Previous research has shown that object recognition involves the rapid transformation of visual information to semantics to be able to accurately identify objects. It has also been shown that object recognition is influenced by the environment, with a congruent environment facilitating object recognition. However, what has not been established is how object recognition is influenced by the environment when the objects are embedded in the real-world and how this impacts the temporal dynamics of object recognition. Through the combination of mEEG, AR, and advanced statistical and computational techniques, we aim to elucidate the impact of real-world environments on the temporal dynamics of object recognition.

## Study status

The study is currently at the start of the data collection phase.

## Data availability

### Underlying data

No data are associated with this article.

### Extended data

Open Science Framework: Context effects on object recognition in real-world environments. https://doi.org/10.17605/OSF.IO/96PNK ( Nicholls, 2022).

Data are available under the terms of the Creative Commons Zero "No rights reserved" data waiver (CC0 1.0 Public domain dedication).
