# Supplementary material for: Context effects on object recognition in real-world environments: A study protocol
Source: Wellcome Open Res. 2023 Jul 14;7:165. Originally published 2022 May 26. [Version 3] doi: 10.12688/wellcomeopenres.17856.3 (PMC10238820; doi:10.12688/wellcomeopenres.17856.3)
Supplement: Peer review history

# Peer review: Reviewer response for version 3

**DOI:** 10.21956/wellcomeopenres.21833.r62941

**Published:** 2023-07-28

**Authors:** Karen L Campbell

**Affiliations:** 1 Department of Psychology, Brock University, Saint Catharines, Ontario, Canada

All of my concerns have been addressed, apart from my final suggestion of a control experiment (which is not necessary for the current proposal).

Is the study design appropriate for the research question?

Partly

Is the rationale for, and objectives of, the study clearly described?

Yes

Are sufficient details of the methods provided to allow replication by others?

Yes

Are the datasets clearly presented in a useable and accessible format?

Yes

Reviewer Expertise:

Cognitive neuroscience

I confirm that I have read this submission and believe that I have an appropriate level of expertise to confirm that it is of an acceptable scientific standard.

# Peer review: Reviewer response for version 3

**DOI:** 10.21956/wellcomeopenres.21833.r62942

**Published:** 2023-07-28

**Authors:** Moreno I. Coco

**Affiliations:** 1 Sapienza University of Rome, Rome, Italy

I thank the authors for their amendments and revisions to the study protocol. I am happy with it and wish them good luck with their next developments.

Is the study design appropriate for the research question?

Partly

Is the rationale for, and objectives of, the study clearly described?

Partly

Are sufficient details of the methods provided to allow replication by others?

Partly

Are the datasets clearly presented in a useable and accessible format?

Not applicable

Reviewer Expertise:

Human cognitive neuroscience, electrophysiology, eye-tracking, visual cognition, statistical modeling

I confirm that I have read this submission and believe that I have an appropriate level of expertise to confirm that it is of an acceptable scientific standard.

# Peer review: Reviewer response for version 3

**DOI:** 10.21956/wellcomeopenres.21833.r62940

**Published:** 2023-07-19

**Authors:** Assaf Harel

**Affiliations:** 1 Department of Psychology, Wright State University, Dayton, OH, USA

Is the study design appropriate for the research question?

Yes

Is the rationale for, and objectives of, the study clearly described?

Yes

Are sufficient details of the methods provided to allow replication by others?

Yes

Are the datasets clearly presented in a useable and accessible format?

Yes

Reviewer Expertise:

Cognitive neuroscience

I confirm that I have read this submission and believe that I have an appropriate level of expertise to confirm that it is of an acceptable scientific standard.

# Peer review: Reviewer response for version 2

**DOI:** 10.21956/wellcomeopenres.20416.r57816

**Published:** 2023-06-02

**Authors:** Karen L Campbell

**Affiliations:** 1 Department of Psychology, Brock University, Saint Catharines, Ontario, Canada

The authors are proposing a novel study that will use mobile electroencephalography (mEEG) and augmented reality (AR) to examine the effects of context on object recognition in a real-world, immersive environment. Participants will move around indoor and outdoor contexts and objects will be encountered in either congruent or incongruent locations. The authors plan to use ERPs (N300/400) and RSA to examine the effects of context on visual and semantic processing, with the expectation that they will observe 1) larger N300/N400 effects for objects in incongruent contexts than those in congruent contexts, and 2) RSA effects for semantic features that are shifted forward in time for congruent objects. Eye movements will also be recorded, and the authors expect longer fixation times for incongruent objects.

 Overall, I thought this was an interesting proposal that aims to further our understanding of how objects are processed in a real-world environment. While I have a few suggestions for improving the design, the authors should be commended for their ambitious plan. Studying the brain under naturalistic conditions is challenging and this proposal attempts to balance experimental control (i.e., preselected objects, controlled onset times, manipulation of context) with an immersive experience that provides improved external validity. Below, I outline my suggestions for the design.

1. One the biggest issues is the lack of counterbalancing of objects across conditions. It is my understanding that one set of objects will serve as congruent objects, while the other set will serve as incongruent. However, any differences observed between the conditions could be attributed to differences between the objects, rather than their congruence with the environment. A tighter design would counterbalance objects across conditions (e.g., ½ the participants see a kettle in the kitchen, while the other ½ see a kettle in the parking lot).
2. Related to this, the authors say that the images in each block will be matched on a number of predictors, but it is unclear why the blocks need to be matched. Shouldn’t the conditions be matched (congruent vs incongruent)? Or are the conditions presented in a blocked fashion?
3. Some more justification could be provided for the exploratory analysis to investigate congruency effects across all electrodes from zero to 1000ms. I think this analysis is warranted, but the authors should explicitly say what will be gained.
4. The purpose of the eye-tracking analyses is unclear since participants are instructed to maintain fixation on the object until prompted to rate how unexpected it was. If participants are supposed to maintain fixation, it seems unlikely that fixation will vary with congruency. The authors may want to consider using pupil dilation at object onset instead, as this may provide a more meaningful measure. Increased pupil dilation is often associated with increased attention and arousal, and thus, you might expect greater pupil dilation for incongruent than congruent trials.
5. Finally, the difference between congruent and incongruent object processing could be due to a speed-up for congruent objects and/or a slow-down for incongruent objects, relative to a context-free condition (more akin to standard laboratory tasks). The authors may want to consider running a control experiment using the same objects appearing on a computer screen while EEG and eye-movements are recorded, against which they could compare their AR data. This would help emphasize what is gained from measuring object processing within a real-world environment and determine whether the difference between congruent and incongruent conditions is due to a benefit from the congruent context or a cost from the incongruent one (and these effects may dissociate for visual and semantic information).

Is the study design appropriate for the research question?

Partly

Is the rationale for, and objectives of, the study clearly described?

Yes

Are sufficient details of the methods provided to allow replication by others?

Yes

Are the datasets clearly presented in a useable and accessible format?

Yes

Reviewer Expertise:

Cognitive neuroscience

I confirm that I have read this submission and believe that I have an appropriate level of expertise to confirm that it is of an acceptable scientific standard, however I have significant reservations, as outlined above.

# Peer review: Reviewer response for version 2

**DOI:** 10.21956/wellcomeopenres.20416.r53523

**Published:** 2023-02-07

**Authors:** Assaf Harel

**Affiliations:** 1 Department of Psychology, Wright State University, Dayton, OH, USA

The authors have fully addressed my concerns. I have no further comments.

Is the study design appropriate for the research question?

Yes

Is the rationale for, and objectives of, the study clearly described?

Yes

Are sufficient details of the methods provided to allow replication by others?

Yes

Are the datasets clearly presented in a useable and accessible format?

Yes

Reviewer Expertise:

Cognitive neuroscience

I confirm that I have read this submission and believe that I have an appropriate level of expertise to confirm that it is of an acceptable scientific standard.

# Peer review: Reviewer response for version 1

**DOI:** 10.21956/wellcomeopenres.19772.r51142

**Published:** 2022-08-08

**Authors:** Assaf Harel

**Affiliations:** 1 Department of Psychology, Wright State University, Dayton, OH, USA

The current study aims to investigate how the semantic background (‘context’) in which objects are embedded impacts their processing, and specifically how this process is substantiated at the neural level. This is not a novel question in and of itself, yet the main conceptual advance of the study is it asking how the impact of semantic context is achieved in real-world settings, that is, with freely behaving participants in naturalistic environments. To address this question, the study proposes to record EEG and eye movements from participants who will be moving in real-world environments while presented with AR-objects varying in their semantic congruency with the surrounding backgrounds. The authors hypothesize that similar to ERP studies using static ‘snapshot’-like stimuli, the current study would also result in N300-400 effects of congruency, including similar behavioral and eye-tracking results.

 This is a sound research project, strongly grounded in previous research and theory and it presents a robust analytical and methodological approach. The study will resolve a concrete theoretical question, namely, whether object-background context effects repeatedly reported in the last forty years can also be observed in naturalistic environments and in the process, will contribute to the advancement of the nascent field of real-world cognitive neuroscience. I do not have any major comments about the study’s experimental design or its data analysis approach.

 However, I do have a small number of comments and suggestions that the authors should consider as ways of improving the study’s overall validity and implications for future research.

 First and foremost, the authors should think about the greater significance of the current study beyond just the question of “will the same effects observed in passive vision will also be observed in active vision conditions?”. I do not mean this as a disparagement, as it is very important to establish the link between lab-based findings and those from more realistic settings. However, if - as the authors suggest - the same results are expected in both modalities, wouldn’t that actually establish the usefulness and veracity of the passive approach to visual perception? Of course, I understand the need to make hypotheses regarding novel paradigms based on previous literature, but the authors should also emphasize what new, unique perspectives could be gained from their novel design. What can be different between the two settings? In other words, the authors should consider a-priori, what free exploration of the environment adds to the phenomenon of semantic context facilitation? It is not just the fact that it’s “real”. As various embodied theories of cognition suggest (just a few examples) - movement in the world, accumulated information over different viewpoints, should all add to the formation of a contextual gist. Why not use the high temporal resolution of the current approach (combining the strength of controlled environments, EEG, and eye-tracking) to provide novel insights into context effects? How does context form? How does it break down? These avenues should be further explored.

 Second, if I understand correctly, the participants will be moving around real-world environments while looking at AR objects that will either be congruent of incongruent with the semantic content of the surrounding environments. In that case, I have a few comments regarding the experimental design.

1. How is the background-object context defined here? Usually, studies of semantic congruency use specific semantic scene categories (often basic-level scene categories), which have distinct objects related to them (i.e., a kitchen and a pan). However, if the background here is defined as a general manmade background (I could tell an office/lab space from Figure 2), how will specific objects be related to the background? Indeed, it may be argued that two manmade tools may be more related to a general manmade background than natural objects, but the level of relatedness here is quite coarse and does not provide a sufficient level of information, and it is definitely not comparable to previous studies of the topic. The authors should be aware of this major limit, or try to think of ways of circumventing it, such as using full VR environments, or have participants explore real spaces (e.g., kitchens, bedrooms, etc.). Also, how are outdoor (I am assuming mostly) natural contexts defined? Those may prove to be quite different from manmade (indoor) scenes. The authors should consider explicitly testing the specific effect of having superordinate scene categories, and how would those, in turn, differ from basic-level ones.
2. Another limit that should be acknowledged by the authors is that AR objects are in themselves segregated from their background, that is, they are probably NOT perceived as real, authentic objects. Thus, even if semantic congruency effects will be observed, the conclusions should be qualified and limited to AR-like circumstances. Otherwise, the authors would first have to establish that real-world objects are perceived and recognized in a similar fashion to AR objects.
3. Third, the current task used by the authors at the end of each trial may induce semantic consistency effect artifactually. Asking participants at the end of each trial whether the object is consistent or not with its background will raise the participants’ awareness of the experimental manipulation, and thus the observed effects may be due to the task manipulation rather than anything else. I would strongly suggest the authors either use this task as is (to get potential ratings of congruency), but then test another sample of participants without using such an explicit task, or simply revert to using a paradigm that does not have an explicit judgment of object congruency.

Is the study design appropriate for the research question?

Yes

Is the rationale for, and objectives of, the study clearly described?

Yes

Are sufficient details of the methods provided to allow replication by others?

Yes

Are the datasets clearly presented in a useable and accessible format?

Yes

Reviewer Expertise:

Cognitive neuroscience

I confirm that I have read this submission and believe that I have an appropriate level of expertise to confirm that it is of an acceptable scientific standard, however I have significant reservations, as outlined above.

# Peer review: Reviewer response for version 1

**DOI:** 10.21956/wellcomeopenres.19772.r51141

**Published:** 2022-06-30

**Authors:** Moreno I. Coco

**Affiliations:** 1 Sapienza University of Rome, Rome, Italy

This prospective study aims to investigate the impact of more realistic contexts on the integration of object-scene through the combined use of augmented reality, mobile electroencephalography, and eye-tracking. Participants (34) will navigate two possible environments (1 indoor, 1 outdoor) which will contain 80 objects, half of which are congruent and the other half incongruent with the environment (e.g., a pen vs. a flower on a desk). Based on the literature the authors expect a larger negative shift of brain activity to be associated with an incongruent compared to congruent object at a latency of 300/400 ms (N300/N400) after having inspected it for the first time. Congruent objects should also be better recognized.

 Overall, I like the study and I believe that it is indeed worth extending research on object recognition, and phenomena of context integration, to more ecological and naturalistic scenarios. There are still some obscurities, though, in aspects of the rationale/background/objectives, the design of the study, and the methods of analysis that I believe the authors could clarify/strengthen.  

 In the rationale, the authors claim that “little is known about what aspects of the recognition process are modulated by the context”. They then cite a couple of studies, most using stimuli that are photoshopped (except, Vo & Wolfe, 2013). Studies collaborators and I have conducted made use of naturalistic visual scenes, demonstrating precise temporal effects of semantic inconsistency on eye-movement responses of younger and older adults (Borges et al., 2020) ^1^ and also uncovered the associated neural activity (Coco et al., 2020 and see also Coco et al., 2017 for an extension to classic findings by Mudrik et al., 2010) ^2^^,^^3^^,^^4^. I struggled to see what an augmented reality task could offer beyond these findings. A different latency? A reduction of the effect? The involvement of different brain areas as the task implies motor actions? A replication of effect and so what’s the advantage of this sophisticated method?

 When continuing to read the protocol, the novelty seems to be the use of RSA but it remains rather vague as to what insights are we supposed to learn, and what aspects of the object-scene integration would it help explain. So, in a way, the objectives could be much better stressed and identified. Some ideas seem to be present in the hypotheses section, but they certainly need expansion and greater clarity (e.g., why would visual and semantic information be represented earlier for congruent objects? And earlier concerning what exactly? A precise time-point of the exploration?)

 The design seems a little weird to me and there are also aspects of the analysis that need to be clarified.

- Only two environments will be used, and then, there will be some superposition of 80 object pictures (40 congruent/40 incongruent) taken from the (Hovhannisyan et al., 2021) ^5^ dataset. If there are only two environments, how would they make sure that objects truly are congruent or incongruent with them? How many objects would fit an office desktop, for example, and how is established the magnitude of the semantic association with it? What about the location? Would it be counterbalanced, or would the object appear always at the same location? And if the objects change position, how would they account for the effects of spatial locations on the likelihood of an object being there? In a nutshell, it is unclear how would the semantic consistent/inconsistency effect be operationalized and properly normed.
- 34 participants sample size based on power analysis of Draschkow et al., (2018) ^6^. This study had 40 participants, and what about the number of items? They seem to have twice as many items, and as the authors are certainly aware, EEG is very noisy, I can only imagine how much noisier would a mEEG be. So, why so few items per participant, which connects also to why so few environments. Note, also, power analysis should be run according to the analysis they wish to do. They propose to use linear mixed effect models, and so they may want to estimate their effect size according to this (e.g. Green & Macleod, 2016) ^7^.
- Eye-tracking: if the sampling rate is 30Hz, how could a speed-velocity saccade detector be reliably used? It is simply too low to be confident about it. Better to use other methods that estimate fixation and saccade on spatial information (e.g., Hessels et al., 2017) ^8^.
- Is the analysis of EEG time-locked to eye-movement information? If so, when and how? The study protocol is very vague about the alignment between all those different signals they are collecting. In general, I felt that the different paragraphs seem to stand alone, so the reader is missing the logical flow connecting the data being analyzed.

Signed

 Moreno I. Coco

Is the study design appropriate for the research question?

Partly

Is the rationale for, and objectives of, the study clearly described?

Partly

Are sufficient details of the methods provided to allow replication by others?

Partly

Are the datasets clearly presented in a useable and accessible format?

Not applicable

Reviewer Expertise:

Human cognitive neuroscience, electrophysiology, eye-tracking, visual cognition, statistical modeling

I confirm that I have read this submission and believe that I have an appropriate level of expertise to confirm that it is of an acceptable scientific standard, however I have significant reservations, as outlined above.

## References

[1] : Age-related differences during visual search: the role of contextual expectations and cognitive control mechanisms. *Neuropsychol Dev Cogn B Aging Neuropsychol Cogn* .27(4) : 10.1080/13825585.2019.1632256 489-516 10.1080/13825585.2019.1632256 31307316

[2] : Disentangling stimulus plausibility and contextual congruency: Electro-physiological evidence for differential cognitive dynamics. *Neuropsychologia* .96: 10.1016/j.neuropsychologia.2016.12.008 150-163 10.1016/j.neuropsychologia.2016.12.008 27965026

[3] : Fixation-related Brain Potentials during Semantic Integration of Object-Scene Information. *J Cogn Neurosci* .32(4) : 10.1162/jocn_a_01504 571-589 10.1162/jocn_a_01504 31765602

[4] : ERP evidence for context congruity effects during simultaneous object-scene processing. *Neuropsychologia* .2010;48(2) : 10.1016/j.neuropsychologia.2009.10.011 507-17 10.1016/j.neuropsychologia.2009.10.011 19837103

[5] : The visual and semantic features that predict object memory: Concept property norms for 1,000 object images. *Mem Cognit* .49(4) : 10.3758/s13421-020-01130-5 712-731 10.3758/s13421-020-01130-5 33469881PMC8081674

[6] : No evidence from MVPA for different processes underlying the N300 and N400 incongruity effects in object-scene processing. *Neuropsychologia* .120: 10.1016/j.neuropsychologia.2018.09.016 9-17 10.1016/j.neuropsychologia.2018.09.016 30261162

[7] : SIMR : an R package for power analysis of generalized linear mixed models by simulation. *Methods in Ecology and Evolution* .2016;7(4) : 10.1111/2041-210X.12504 493-498 10.1111/2041-210X.12504

[8] : Noise-robust fixation detection in eye movement data: Identification by two-means clustering (I2MC). *Behav Res Methods* .2017;49(5) : 10.3758/s13428-016-0822-1 1802-1823 10.3758/s13428-016-0822-1 27800582PMC5628191
